# The Germ of Texas Fever

**Published:** 1891-03

**Authors:** R. R. Dinwiddie

**Affiliations:** Veterinarian; Arkansas Agricultural Experiment Station


### THE GERM OF TEXAS FEVER.

### CAN IT BE CULTIVATED FROM THE ORGANS OF SOUTHERN CATTLE?

BY R. R. DINWIDDIE, VETERINARIAN.
*Arkansas Agricultural Experiment Station.*

During the past summer I have devoted my time to experimental studies of the blood and viscera of cattle, living within the regions of the South permanently infected with the virus of Texas Fever, and herewith briefly present the results of this portion of my work as a contribution to the literature of the subject. My object has been the determination of this question:—Do cattle from the South, infection bearing cattle, contain the agent of infection, in their *systems,* as distinct from the intestinal tract or any external medium in which the virus may be conveyed?

I have assumed, for the purposes of this work, that the causative agent of Texas Fever is a Schizophyte capable of cultivation or demonstration by the usual methods of bacterial investigation.

Those who have acquainted themselves with the reports of recent researches on Texan Fever will recognize the basis for this assumption as well as the reason for looking for the germ in this situation.

Bacteriologists now admit that the living tissues of the healthy animals do not, except in rare instances, contain bacterial germs. If then, as has been asserted and very widely accepted, there is constantly present in the liver, spleen, kidneys and blood of native Southern cattle any bacterial organism, we have good grounds for inferring this to be the much sought-for germ of Texas Fever, and this would be the most suitable place for obtaining it in pure cultivations.

My investigations were carried on at Little Rock, Arkansas, which with the surrounding country is infected territory. Cattle shipped there from the North die of Texas Fever to the extent of at least sixty per cent. The material was obtained at the slaughter houses of that city from cattle raised in the vicinity or coming from points further south.

In order to determine the presence of bacteria in tissues or in fluids, two methods are available; these are microscopic examination of the material and inoculation of culture media in sterile tubes. It is possible that bacteria may be present even if both of these methods fail to give positive results and hence negative evidence in bacteriology is evidence only and not proof, but, on the other hand, until their presence has been so proved we are not justified in assuming their existence, however well such assumption may harmonize with our preconceived theories. My methods of work are such as are employed in modern bacterial research. The culture media employed were beef-infusion peptone, nutrient agar, glycerine agar, milk, and cooked potato all contained in test tubes of varying sizes. Inoculations were made by means of the platinum wire, with the usual precautions necessary to prevent contamination, and conducted both in the slaughter-house and in the laboratory. In the former case the material was used within half an hour after death, immediately after death in the case of the fluids.

Material for use in the laboratory was wrapped in cloths saturated in sublimate solution, or conveyed in sterile sealed pipettes, and examinations and inoculations of tubes made from same as early as possible, generally within three hours after death.

In all, during the summer, I made in this manner eight series of inoculations from as many animals. In each series a large number of tubes were employed, generally forty to sixty, in order to diminish the risk of forming conclusions erroneous owing to accidental contaminations. A certain number of tubes were kept in the thermostadt at 35° C., the remainder at ordinary room temperature in summer.

The result of these investigations force me to come to conclusions at variance with others who have preceded me in this field of work.

With one exception which I shall presently refer to, the blood, liver and spleen of these cattle failed to give rise to any growth when transferred by the needle to culture media of any kind.

I have frequently, by means of the looped wire, transferred to beef infusion tubes sufficient blood to distinctly tinge the fluid, which nevertheless remained otherwise clear after two or three weeks in the incubator.

Spleen pulp was also in all cases sterile. Inoculations from the bile were not always followed by results admitting of such ready interpretations. In the majority of cases tubes sowed from this source gave rise to no growth but in others I did obtain cultures of micro-cocci. Whether these growths in all cases came actually from the bile or were contaminations I cannot at this time positively affirm. Some cultures from this source were manifestly mixed. I am inclined to think that the bile in the gall-bladder of cattle at least is usually free from germs but may at times contain diverse organisms by migration or growth of the latter from the intestinal canal.

The exceptional case referred to above was the first animal from which cultures were attempted. In seventy-five per cent. of tubes inoculated from the liver of this case growths were obtained of a micro-coccus. These growths occurred alike in tubes inoculated in the slaughter-house and in the laboratory, and I have little doubt but that they really had their source from germs contained in the liver, although that organ appeared to be in a normal condition. None of the other materials made use of in this animal gave rise to growths except the bile which gave a mixed culture of the same micro-coccus along with another of somewhat similar microscopic appearance but differing in its growth on the various media.

The organism derived from the liver occurred in pairs and in short chains of not more than four or five elements. It is refrangent, exhibits active molecular motion in fluids, and is readily stained. Diameter 1.6 y. It grows readily on all media employed, at room temperature and in thermostadt. In beef infusion it clouds the fluid in twenty-four hours, the opacity extending in streaks downwards; a sediment soon forms; there is no surface pellicle.

On agar the growth is pure white, raised and limited, with smooth surface and margins. It does not spread over the surface. Gelatine is not fluidified.

I do not further enlarge on the biology of this germ as I consider it to have no pathogenic significance. It was never encountered again in the organs of cattle, but later I did succeed in isolating apparently the same form from water of the range on which these cattle grazed. Sometime later the opportunity occurred of testing its effects on animals. Inoculation and feeding experiments on calves and guinea-pigs gave negative results. I therefore conclude that the liver of cattle may occasionally harbor, perhaps temporarily and in greater or less quantity, one or more species of bacterial organisms derived from the food or water, without producing any deleterious effects.

Such is the result of my culture experiments. Briefly, I may add that microscopic examination of the same materials have not enabled me to confirm the results of others who have found bacteria in similar situations. Neither in smear cover-cover glass preparations nor in sections from organs of Southern cattle have I been able by any method of staining to demonstrate the presence of bacteria to my own satisfaction.

As every established fact bearing on the etiology of Texas Fever has its importance I will advance this as the result of my investigations, and in answer to the question which forms the title for this paper, namely:

*That healthy Southern cattle raised on infected pastures do not regularly bear in their tissues, blood or viscera any bacterial organism capable of demonstration by ordinary microscopical methods, or of growth on the ordinary culture media employed in bacterial research.*

